# Palliative Radiotherapy for Leptomeningeal Carcinomatosis–Analysis of Outcome, Prognostic Factors, and Symptom Response

**DOI:** 10.3389/fonc.2018.00641

**Published:** 2019-01-08

**Authors:** Rami A. El Shafie, Karina Böhm, Dorothea Weber, Kristin Lang, Fabian Schlaich, Sebastian Adeberg, Angela Paul, Matthias F. Haefner, Sonja Katayama, Juliane Hörner-Rieber, Philipp Hoegen, Sarah Löw, Jürgen Debus, Stefan Rieken, Denise Bernhardt

**Affiliations:** ^1^Department of Radiation Oncology, Heidelberg University Hospital, Heidelberg, Germany; ^2^National Center for Radiation Oncology, Heidelberg Institute for Radiation Oncology, Heidelberg, Germany; ^3^Institute of Medical Biometry and Informatics, Heidelberg University Hospital, Heidelberg, Germany; ^4^Heidelberg Ion-Beam Therapy Center (HIT), Heidelberg University Hospital, Heidelberg, Germany; ^5^Department of Neurology, Heidelberg University Hospital, Heidelberg, Germany; ^6^Clinical Cooperation Unit Radiation Oncology (E050), German Cancer Research Center (DKFZ), Heidelberg, Germany; ^7^Deutsches Konsortium für Translationale Krebsforschung (DKTK), Partner Site Heidelberg, German Cancer Research Center (DKFZ), Heidelberg, Germany

**Keywords:** leptomeningeal metastases, carcinomatous meningitis, neurologic function, palliative, radiotherapy, whole-brain radiotherapy, craniospinal irradiation

## Abstract

**Introduction:** The purpose of this article is to report our institution's 10-year experience on palliative radiotherapy for the treatment of leptomeningeal carcinomatosis (LC), assessing survival, neurologic outcome, and prognostic factors.

**Patients and methods:** We retrospectively analyzed 110 patients who received palliative radiotherapy for LC between 2008 and 2018. The most common histologies were breast cancer (*n* = 43, 39.1%) and non-small cell lung cancer (NSCLC) (*n* = 31, 28.2%). Radiotherapy was administered as whole-brain radiotherapy (WBRT) (*n* = 51, 46.4%), focal spinal RT (*n* = 11, 10.0%) or both (*n* = 47, 42.7%). Twenty-five patients (22.7%) were selected for craniospinal irradiation. Clinical performance and neurologic function were quantified on the neurologic function scale (NFS) before and in response to therapy. A Cox Proportional Hazards model with univariate and multivariate analysis was fitted for survival.

**Results:** Ninety-eight patients (89.1%) died and 12 (10.9%) were alive at the time of analysis. Median OS from LC diagnosis and from the beginning of RT was 13.9 weeks (IQR: 7.1–34.0) and 9.9 weeks (IQR: 5.3–26.3), respectively. In univariate analysis, prognostic of longer OS were a Karnofsky performance scale index (KPI) of ≥70% (HR 0.20, 95%-CI: [0.13; 0.32], *p* < 0.001), initially moderate neurological deficits (NFS ≤2) (HR 0.32, 95% CI: [0.19; 0.52], *p* < 0.001), symptom response to RT (HR 0.41, 95%-CI: [0.26; 0.67], *p* < 0.001) and the administration of systemic therapy (HR 0.51, 95%-CI: [0.33; 0.78], *p* = 0.002). Prognostic of inferior OS were high-grade myelosuppression (HR 1.78, 95% CI: [1.06; 3.00], *p* = 0.03) and serum LDH levels >500 U/l (HR 3.62, 95% CI: [1.76; 7.44], *p* < 0.001). Clinical performance, symptom response and serum LDH stayed independently prognostic for survival in multivariate analysis. RT was well-tolerated and except for grade III myelosuppression in 19 cases (17.3%), no high-grade acute toxicities were observed. Neurologic symptom stabilization was achieved in 83 cases (75.5%) and a sizeable improvement in 39 cases (35.5%).

**Conclusion:** Radiotherapy is a well-tolerated and efficacious means of providing symptom palliation for patients with LC, delaying neurologic deterioration while probably not directly influencing survival. Prognostic factors such as clinical performance, neurologic response and serum LDH can be used for patient stratification to facilitate treatment decisions.

## Introduction

Leptomeningeal carcinomatosis (LC), as defined by the current EANO-ESMO guidelines, refers to a usually multifocal spread of tumor cells to the leptomeninges (pia or arachnoidea) and/or the cerebrospinal fluid (CSF) (1). Incidence amounts to approximately 5–10% of patients presenting with metastatic cancer (1–3). The most common histologies to develop LC are breast cancer (12–35%), lung cancer (10–26%), and melanoma (5–25%). The overall incidence of LC shows an upward tendency, possibly attributable to an increase in the availability of efficacious systemic therapies and consequently prolonged survival. Advances in imaging and diagnostic capabilities may also contribute to an increasing number of LC diagnoses (4–7).

Clinical presentation can vary among a wide range of neurological symptoms, most frequently headaches and nausea (25–39%), motoric or sensory dysfunction (17–21%), altered mental status (16%) or cranial nerve deficits (11–14%) (5, 8). Notably, in 50–80% of the cases, LC is associated with additional parenchymal brain metastases (5, 8–10).

With reported median overall survival (OS) of 10–15 weeks, prognosis is dismal and therapeutic options are limited. Several approaches have been established to provide symptom palliation or prolong survival for selected subgroups (8, 11–13). Frequently, focal radiotherapy of symptomatic spinal lesions is performed to preserve neurologic function and control symptoms (13). This approach is often combined with whole brain radiotherapy (WBRT), which can positively affect survival, especially in the presence of additional brain metastases (13, 14). In addition to supportive corticosteroids, medicamentous treatment has been established primarily in the form of intrathecal chemotherapy (15–17). With the rising diversity of available small-molecule targeted therapies and the advances in molecularly informed decision-making, efficacy in the CNS has been evident for several of those drugs (18, 19).

While providing questionable benefit, many of the abovementioned therapeutical approaches are associated with substantial toxicity, most frequently critical myelosuppression and potential worsening of pre-existing symptoms (20, 21). Consequently, careful clinical assessment and patient selection, as well as interdisciplinary decision-making, are warranted before determining a course of action. It is the aim of this report to discuss our institution's experience over a period of 10 years with the radiotherapeutical treatment of 110 patients with LC and evaluate outcome, toxicity, and predictive clinical factors.

## Patients and Methods

To assess the palliative outcome and efficacy of radiotherapy (RT) in the setting of LC, we performed a retrospective analysis of 110 patients, who received radiotherapy for LC between 2008 and 2018 at our institution. Patient data were extracted from an oncologic research database maintained at our institution, as well as from the patients' detailed medical records. All reviews were performed following institutional guidelines and the Declaration of Helsinki of 1975 in its most recent version. Ethics approval for the study and a waiver of written informed consent was granted by the Heidelberg University ethics committee on April 12th, 2018 (#S-172/2018). Patient confidentiality was maintained by anonymizing patient data to remove any identifying information.

### Patient Characteristics

Median patient age at LC diagnosis was 59 years, and median interval from primary diagnosis was 29.5 months. Detailed patient characteristics are illustrated in Table 1. Primary histology varied: Breast cancer was most frequent (*n* = 43, 39.1%), followed by lung cancer (*n* = 31, 28.2%).

**Table 1:** Patient characteristics.

**AGE(YEARS)**	
Mean	57.9
SD	12.27
Median	59
Q1–Q3	52–68
Min.–Max.	17.0–78.0
**GENDER**	
Female	63 (57.3%)
Male	47 (42.7%)
**PRIMARY HISTOLOGY**	
Breast cancer	43 (39.1%)
Lung cancer	31 (28.2%)
Gastrointestinal cancers	9 (8.2%)
Melanoma	7 (6.4%)
Prostate cancer	6 (5.5%)
Others	14 (12.7%)
**INTERVAL FROM PRIMARY DIAGNOSIS TO LC DIAGNOSIS (MONTHS)**	
Mean	60.4
SD	76.81
Median	29.5
Q1–Q3	10–86
Min.–Max.	0.0–459.0
**KPI AT LC DIAGNOSIS (%)**	
Mean	60
SD	14
Median	60
Q1–Q3	60–70
Min.–Max.	30–90
**METASTASES OUTSIDE CNS**	
Yes	82 (74.5%)
No	28 (25.5%)
**PARENCHYMAL BRAIN METASTASES**	
Yes	82 (74.5%)
No	28 (25.5%)
**LAST THERAPY BEFORE RT**	
None	33 (30.0%)
Systemic chemotherapy	30 (27.3%)
Targeted therapy	20 (18.2%)
Antihormonal therapy	11 (10.0%)
Intrathecal chemotherapy	9 (8.2%)
Combination therapy	7 (6.3%)
**THERAPY AFTER RT**	
None	68 (61.8%)
Systemic chemotherapy	16 (14.5%)
Targeted therapy	14 (12.7%)
Antihormonal therapy	5 (4.5%)
Intrathecal chemotherapy	4 (3.6%)
Combination therapy	3 (2.7%)
**DIAGNOSTICS**	
MRI	110 (100.0%)
Lumbar puncture	64 (58.2%)
Both	64 (58.2%)
**LC SPREAD**	
Spine	65 (59.1%)
Brain	79 (71.8%)
Both	46 (41.8%)
**NEUROLOGIC FUNCTION (NFS) BEFORE TREATMENT**	
0	4 (3.6%)
1	26 (23.6%)
2	57 (51.8%)
3	23 (20.9%)
**LDH LEVEL (U/L)**	
Mean	371.9
SD	217.03
Median	315
Q1–Q3	245.75–419
Min.–Max.	134.0–1267.0
**CRP LEVEL (MG/L)**	
Mean	19.4
SD	37.53
Median	3.2
Q1–Q3	1.9–15.6
Min.–Max.	1.9–249.5
**HEMOGLOBIN LEVEL (U/L)**	
Mean	12.5
SD	1.92
Median	12.9
Q1–Q3	11.2–13.9
Min.–Max.	7.9–16.1
**RPA CLASS (82 PATIENTS WITH BRAIN METASTASES)**	
1	11 (13.4%)
2	29 (35.4%)
3	42 (51.2%)
**GPA SCORE (82 PATIENTS WITH BRAIN METASTASES)**	
0–1	71 (64.5%)
1.5–2.5	10 (9.1%)
3.0	1 (0.9%)
3.5–4.0	0 (0.0%)

*LC, leptomeningeal carcinomatosis; CNS, central nervous system; RT, radiotherapy; KPI, Karnofsky performance scale index; MRI, magnetic resonance imaging; NFS, neurologic function scale; CRP, C-reactive protein; LDH, lactate dehydrogenase; RPA, recursive partitioning analysis; GPA, graded prognostic assessment*.

Symptoms and overall clinical performance (Karnofsky performance scale index, KPI) at the beginning of RT and during follow-up were extracted from the patients' medical records and quantified regarding symptom control, improvement or worsening after therapy. Based on the documented symptoms, a neurologic function status (NFS) was derived and used to assess the palliative effect achieved by RT (22). Neurologic function was quantified in accordance with the validated functional outcome measure of two past RTOG trials that has since been adopted in several clinical analyses (22–24). It was classified on the five-point NFS scale as follows: asymptomatic (0), minor neurological symptoms (1), moderate neurological symptoms (2), neurologically seriously limited, requiring hospitalization (3), and requiring hospitalization and constant nursing care (4). The outcome of NFS was assessed as either stable, improved or worsened, according to documented symptoms. Symptom control was defined as a constant value of the NFS at therapy completion or first follow-up if available, whereas improvement was defined as a reduction of the NFS by at least one point from baseline. Recursive partitioning analysis (RPA) class and graded prognostic assessment (GPA) score were calculated for all patients who showed parenchymal brain metastases (25, 26). Date of death was obtained from medical and official records. Treatment-related toxicity was rated according to the Common Terminology Criteria for Adverse Events (CTCAE) v. 4.0 (27). Detailed patient characteristics are illustrated in Table 1.

### Treatment

Treatment indication was discussed interdisciplinarily in the context of our institution's comprehensive cancer center. Palliative treatment was indicated for symptomatic spinal lesions. Additionally, if previously untreated parenchymal brain metastases or radiographic intracerebral LC were present, WBRT including the uppermost two cervical vertebrae (WBRT-C2) was performed. Twenty-five patients were selected for craniospinal irradiation (CSI) based on good performance and estimated clinical benefit.

For cranial RT, an individual head fixation mask was fitted for each patient. Treatment planning was performed using a 3 mm computed tomography (CT) and Gadolinium enhanced MR-imaging when available. The most commonly prescribed dose for WBRT was 30 Gy in 10 fractions. An additional dose of most commonly 9 Gy in 3 fractions to large brain metastases was applied after three-dimensional conformal (3DCRT) treatment planning in 9 cases. Treatment was delivered at a linear accelerator using two laterally opposing fields for WBRT and multi-field technique for 3DCRT, as has been previously described (28, 29).

For spinal RT, the symptomatic spinal segments were identified using clinical assessment and Gadolinium enhanced MR-imaging. Treatment planning was performed based on a 3- or 5-mm CT. The target segments, commonly including a safety margin of one vertebra in upward and downward direction, but at least of 5 mm were defined as PTV. Detailed aspects of target volume delineation for spinal irradiation at our institution have been described earlier (28). The most commonly prescribed dose for spinal irradiation was 30 Gy in 10 fractions. Treatment for segmental irradiation was most commonly delivered at a linear accelerator using multi-field technique for 3DCRT. For CSI, if clinically feasible, vertebral bodies were spared to reduce hematologic toxicity. CSI was delivered as helical intensity-modulated radiotherapy (IMRT) at a TomoTherapy machine (Accuray Inc., Sunnyvale, California). All aspects of TomoTherapy planning for CSI have been described earlier (30). Details on treatment parameters are illustrated in Table 2.

**Table 2:** Prior radiotherapy and detailed treatment parameters for current irradiation.

**PRIOR RADIOTHERAPY OUTSIDE CNS**	
No	68 (61.8%)
Yes	42 (38.2%)
**PRIOR RADIOTHERAPY TO PARTS OF THE CNS**	
None	63 (57.3%)
Spinal	23 (20.9%)
SRS	11 (10.0%)
WBRT	16 (14.5%)
Combination of the above	3 (2.7%)
**EXTENT OF CURRENT IRRADIATION**	
Only WBRT	51 (46.4.0%)
Only focal spinal irradiation	11 (10.0%)
Both	47 (42.7%)
Total or subtotal craniospinal irradiation	25 (22.7%)
**PHYSICAL DOSE PER FRACTION FOR CURRENT RT (GY)**	
Mean	2.7
SD	0.73
Median	3.0
Q1–Q3	2.4–3.0
Min.–Max.	2.0–8.0
**PHYSICAL CUMULATIVE DOSE FOR CURRENT RT (GY)**	
Mean	30.3
SD	6.1
Median	30.0
Q1–Q3	30–35
Min.–Max.	8–51.0
**BIOLOGICALLY EQUIVALENT CUMULATIVE DOSE FOR CURRENT RT (GY)**	
Mean	38.2
SD	7.19
Median	39
Q1–Q3	39–42.5
Min.–Max.	11.7–61.7

Biologically equivalent dose (BED) is calculated with an underlying assumed α/β = 10 for malignant cells. CNS, central nervous system; SRS, stereotactic radiosurgery; WBRT, whole-brain radiotherapy

### Statistical Analysis

For baseline analyses descriptive statistics are used, continuous variables are given as means (SD) and/or median (IQR and range, as appropriate) and categorical variables as absolute and relative frequencies. The quantiles of the follow-up time were calculated using the reverse Kaplan–Meier method (31), additionally the naïve median follow-up over all patients was calculated. OS was calculated independently from the date of LC diagnosis and from the beginning of RT to last follow-up or death. Overall survival (OS) was investigated using the method of Kaplan–Meier. Survival curves for prognostic factors were compared using a two-sided log rank test. To identify prognostic factors on overall survival, univariate and multivariate Cox regression were used. Variables with significance in univariate analysis and meeting statistical quality criteria for multivariate modeling (applicable to all patients, not severely imbalanced) were considered in multivariate Cox regression. Since this was a retrospective exploratory data analysis, *p*-values are of descriptive nature. A descriptive *p*-value of <0.05 was considered as statistically significant. Statistical analyses were performed with the software *R Version 3.4.3*.

## Results

### Survival and Prognostic Factors

**Figure 1:**
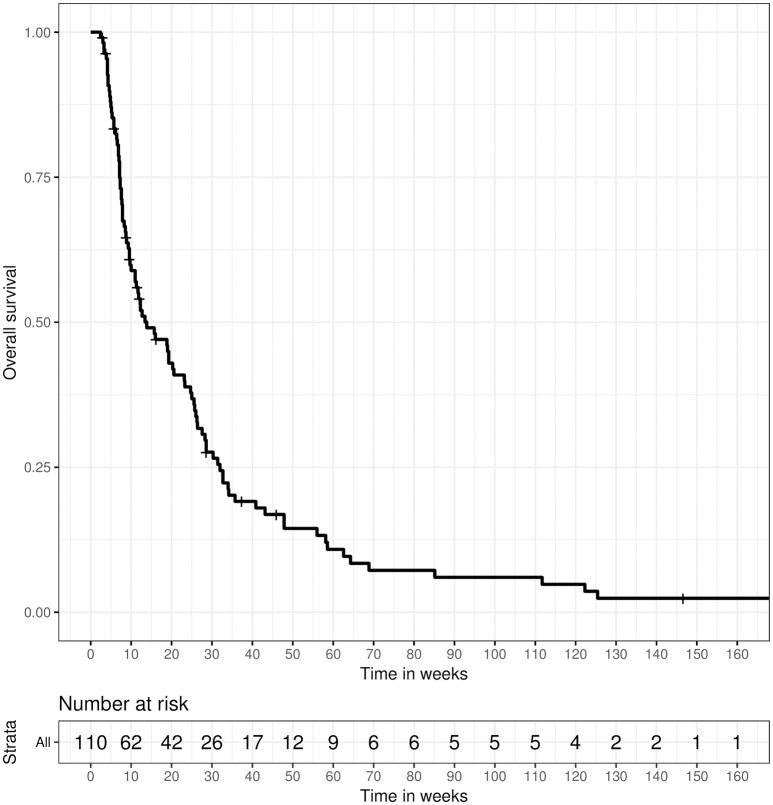
Overall survival for 110 patients receiving radiotherapy for the treatment of LC, calculated from the date of LC diagnosis until death or last follow-up.

The 80%-quantile of the follow-up time, as estimated by the reverse Kaplan-Meier method, was 45.86 weeks (90%-quantile: 16.14 weeks). Directly calculating the naive median follow-up over all patients resulted in a median follow-up of 11.93 weeks (IQR: 7.14–28.57). Ninety-eight (89.1%) patients had died at the time of analysis and 12 (10.9%) patients were still alive. Median overall survival (OS) from LC diagnosis in all treated patients was 13.86 weeks (IQR: 7.14–32.00) (Figure 1). Median OS from the beginning of RT was 9.86 weeks (IQR 5.29–26.29).

**Figure 2:**
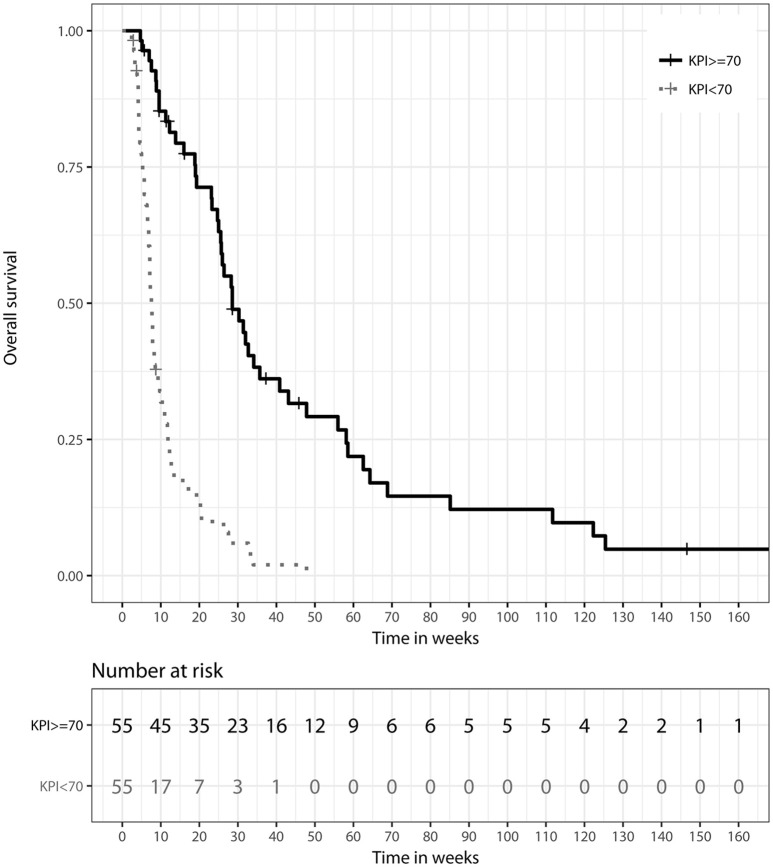
Kaplan–Meier survival curves stratified by Karnofsky performance scale index (KPI) at LC diagnosis, *p* < 0.001 (two-sided log rank test).

A Karnofsky performance scale index (KPI) of ≥70% was associated with a longer median OS of 28.57 weeks (IQR: 19.00–58.14), compared to 7.57 weeks (IQR: 5.29–11.86) for patients with reduced clinical performance and a KPI of <70%. This difference showed significant prognostic relevance in univariate (HR 0.20, 95%-CI: [0.13; 0.32], *p* < 0.0001) and multivariate analyses (HR 0.25, 95%-CI: [0.11; 0.54], *p* < 0.001), as illustrated in Figure 2.

**Figure 3:**
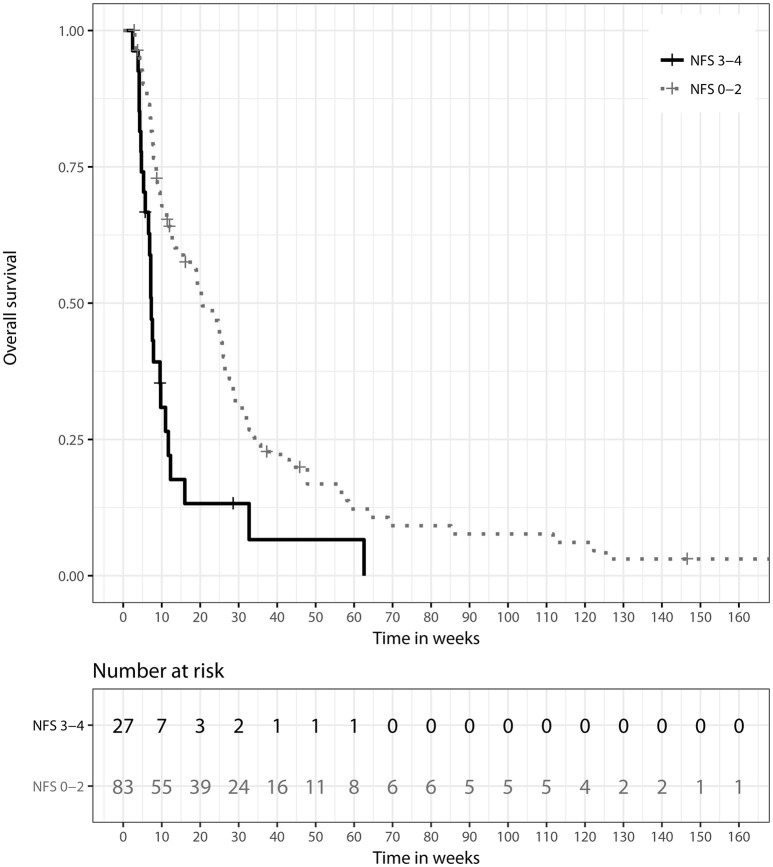
Kaplan–Meier survival curves stratified by neurologic function scale (NFS) response to treatment, *p* = 0.009 (two-sided log rank test).

Patients showing only mild or moderate neurologic symptoms at initial presentation (NFS ≤2) had a longer median OS of 20.57 weeks (IQR: 8.57–35.71) than patients presenting with severe neurologic deficits (NFS > 2). For those patients, median OS was 7.14 weeks (IQR: 4.29–12.71), and prognostic impact of a better NFS score was detectable in univariate analysis (HR 0.32, 95% CI: [0.19; 0.52], *p* < 0.001). Overall symptom response to treatment was defined as a stabilization or improvement of the patient's NFS score at treatment completion or first follow-up. Symptom response was a significant prognostic factor for longer OS in univariate (HR 0.41, 95%-CI: [0.26; 0.66], *p* < 0.001) and multivariate analyses (HR 0.41, 95%-CI: [0.21; 0.80], *p* = 0.009). Respective figures for median OS were 20.57 weeks (IQR: 8.57–35.71) vs. 7.14 weeks (IQR: 4.29–12.71), as illustrated in Figure 3. Patients classified class 3 according to RPA showed significantly inferior OS when compared to class 1 in univariate analysis (HR 4.93, 95%-CI: [2.16–11.25], *p* < 0.001). Comparison of RPA class 2 to RPA class 1 yielded no statistical significance. GPA score showed no significant impact on OS.

**Figure 4:**
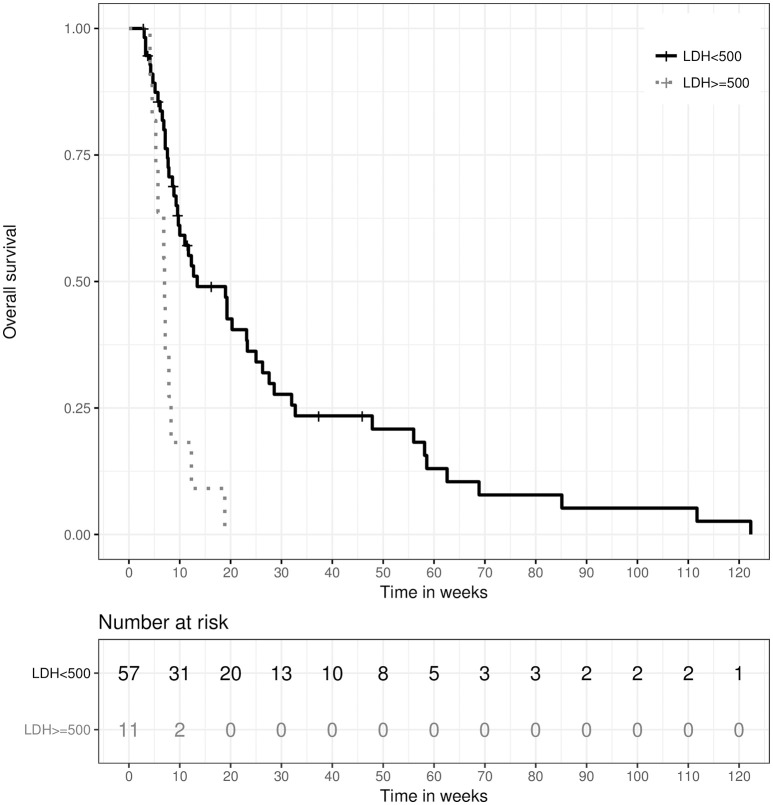
Kaplan–Meier survival curves stratified by serum lactate dehydrogenase (LDH) levels > 500 U/l, *p* = 0.002 (two-sided log rank test).

Baseline inflammatory markers and hemoglobin levels were determined out of bloodwork done within the period of 2 weeks prior to until 3 days after the beginning of RT. Furthermore, patients were regularly screened for the early detection of high-grade radiogenic myelosuppression. As such were considered any one of following: anemia with hemoglobin levels < 8 g/dl, neutropenia with leucocyte levels < 1.0/nl or thrombopenia with thrombocyte levels < 50/nl. In univariate analysis, a lactate dehydrogenase (LDH) level of >500 U/l was strongly associated with inferior median OS of 7.0 weeks (IQR: 5.29–8.29) vs. 13.43 weeks (IQR: 7.57–32.71), HR 3.62, 95%-CI: [1.8; 7.4], *p* < 0.001, as were higher C-reactive protein (CRP) levels >50 mg/l, though significance for CRP was not reached (6.86 weeks (IQR: 4.14–7.57) vs. 18.86 weeks (IQR: 7.71–32.71), HR 1.93, 95%-CI: [0.96; 3.88], *p* = 0.063). In multivariate analysis, LDH >500 U/l stayed an independent prognostic factor for inferior OS (HR 3.59, 95%-CI: [1.61; 8.01], *p* = 0.002), as illustrated in Figure 4. A higher hemoglobin level showed a trend toward longer OS in univariate analysis, but did not reach statistical significance (HR 0.90, 95%-CI: [0.80; 1.01], *p* = 0.079). However, the occurrence of high-grade myelosuppression during RT was significantly associated with inferior OS in univariate analysis (HR 1.78, 95%-CI: [1.06; 3.00], *p* = 0.030).

The application of systemic therapy was assessed before and after radiotherapy, and in univariate analysis, significantly improved OS could be observed in patients who received systemic therapy after the beginning of RT (26.0 weeks (IQR: 9.57–58.14) vs. 11.28 weeks (IQR: 6.86–25.57), HR 0.51, 95%-CI: [0.33; 0.78], *p* = 0.002).

No prognostic significance regarding OS could be detected for any single primary histology, when tested against all other histologies. Similarly, the radiographic extent of leptomeningeal tumor spread, affecting only intracranial, only spinal or both meninges, did not significantly impact OS. Neither did the extent of the radiotherapy field(s), when comparing patients receiving only WBRT, only focal spinal RT or both. No significant difference in OS was detected for the subgroup of 25 patients who received total or subtotal craniospinal irradiation. Additional factors without statistically significant impact on OS were age at LC diagnosis, gender, the presence of parenchymal brain metastases and the application of corticosteroids. All factors examined for univariate and multivariate analyses, corresponding *p*-values and hazard ratios are listed in Tables 3, 4.

**Table 3:** Factors analyzed in univariate cox regression with corresponding hazard ratios and *p*-values.

**Factor analyzed for association with overall survival**	**Hazard ratio**	**95% CI**	***p***
**BASELINE CHARACTERISTICS**			
Age at LC diagnosis	0.718	0.475–1.080	0.115
Gender	1.150	0.766–1.720	0.505
Interval primary diagnosis to LC diagnosis	1.000	0.998–1.000	0.313
Metastases outside CNS present	0.767	0.485–1.210	0.256
Breast cancer histology^*^	0.956	0.635–1.440	0.830
Lung cancer histology^*^	1.160	0.727–1.850	0.533
RPA class 3 (reference = 1)	4.935	2.165–11.250	**<0.001**
RPA class 2 (reference = 1)	1.334	0.592–3.006	0.487
GPA score 1.5–2.5	0.426	0.756–3.096	0.236
**CLINICAL PERFORMANCE**			
Initial KPI	0.203	0.128–0.322	**<0.001**
Initial NFS	0.315	0.192–0.517	**<0.001**
**EXTENT OF CNS METASTASES**			
Intracranial LC	0.944	0.622–1.430	0.786
Spinal LC	0.959	0.600–1.530	0.861
Tumor cells in CSF	1.150	0.768–1.730	0.493
Parenchymal brain metastases	0.856	0.538–1.360	0.512
**TREATMENT AND RESPONSE**			
Systemic therapy before start of RT	0.809	0.521–1.260	0.345
Systemic therapy after start of RT	0.507	0.331–0.777	**0.002**
Only spinal irradiation	0.809	0.406–1.610	0.546
Only WBRT	0.985	0.661–1.470	0.943
Both focal spinal and cranial irradiation	1.080	0.724–1.620	0.697
Total or subtotal craniospinal irradiation	0.804	0.495–1.300	0.376
Prophylactic or therapeutic corticosteroids	1.010	0.552–1.860	0.965
Neurologic response to treatment	0.412	0.255–0.665	**<0.001**
High-grade myelosuppression after RT	1.780	1.060–3.000	**0.030**
**SERUM AND INFLAMMATORY MARKERS**			
CRP > 50 mg/l	1.930	0.964–3.880	0.063
Higher hemoglobin level	0.900	0.801–1.010	0.079
Serum LDH > 500 U/l	3.620	1.760–7.440	**<0.001**

*Significant p-values indicated in bold type*.

* *No single histology reached significance in univariate analysis; for better readability only the two largest subgroups are listed. ^#^Reference group: GPA score ≤ 1; only one patient had a GPA score ≥ 3, consequently this group was not considered. LC, Leptomeningeal carcinomatosis; KPI, Karnofsky performance scale index; CNS, central nervous system; NFS, Neurologic function scale; CSF, cerebrospinal fluid; RT, radiotherapy; WBRT, whole-brain radiotherapy; CRP, C-reactive protein; LDH, lactate dehydrogenase*.

**Table 4:** Factors analyzed in multivariate cox regression with corresponding hazard ratios and p-values.

**Factor analyzed for association with overall survival**	**Hazard ratio**	**95% CI**	***p***
Initial NFS	0.756	0.366–1.561	0.450
Initial KPI	0.246	0.112–0.541	**<0.001**
Neurologic response to treatment	0.407	0.207–0.798	**0.009**
High-grade myelosuppression after RT	1.273	0.608–2.664	0.522
Serum LDH > 500 U/l	3.592	1.611–8.012	**0.002**
Systemic therapy after start of RT	0.738	0.364–1.495	0.399

*Significant p-values indicated in bold type. All variables significant in univariate analysis were included in multivariate modeling. KPI, Karnofsky performance scale index; NFS, Neurologic function scale; RT, radiotherapy; WBRT, whole-brain radiotherapy; CRP, C-reactive protein; LDH, lactate dehydrogenase*.

### Toxicity and Symptom Response

Palliative treatment was adequately tolerated, and overall, acute treatment-associated toxicity was manageable, falling under grades I and II according to CTCAE v4.03. It consisted mainly of fatigue, most commonly observed in 50.9% (*n* = 56) of the patients, and nausea, observed in 30.0% (*n* = 33) of the patients. High-grade myelosuppression, as defined above, occurred in 17.3% (*n* = 19) of the cases and was controllable by medical intervention. Ninety-four patients (85.5%) completed treatment, and in 16 cases (14.5%) treatment was discontinued due to disease-associated general deterioration or patient wish. Prophylactic or therapeutic corticosteroids were administered in 96 cases (87.3%). An overview of acute treatment-related toxicities is presented in Table 5.

**Table 5:** Acute treatment-associated toxicity.

	**Grade I**		**Grade II**		**Grade III**	
**Toxicity**	***n***	**%**	***n***	**%**	***n***	**%**
Nausea	17	15.5	15	13.6	–	–
Fatigue	13	11.8	55	50.0	–	–
Skin erythema	7	6.4	–	–	–	–
High-grade myelosuppression	–	–	–	–	19	17.3

Patients initially presented with a variety of LC-associated symptoms, most common among which were motor and sensory deficits, observed in 76 (69.1%) and 60 (54.5%) patients, respectively. Headaches manifested in 41 (37.3%) and visual impairments in 34 (30.9%) patients. Summarizing pre-existing neurologic symptoms on the five-point neurologic function scale, as described above, most patients (*n* = 83, 75.5%) presented with mild or moderate symptoms (NFS 1-2). Twenty-three patients (20.9%) showed severe neurologic deficits at initial presentation, and 4 patients (3.6%) were asymptomatic. Neurologic symptom response to treatment in the form of NFS score stabilization or improvement, as defined above, could be achieved in a total of 83 cases (75.5%); 39 (35.5%) of those cases showed a sizeable improvement. Twenty-seven patients (24.5%) showed further neurologic deterioration, irrespective of treatment. Detailed information on individual symptoms, as well as NFS, and their respective response to treatment are provided in Table 6.

**Table 6:** Response of individual symptoms and neurologic function scale (NFS) to treatment.

	**Before palliative RT**		**Clinical outcome after palliative RT**					
**Clinical symptoms and NFS**			**Improvement**		**Stabilization**		**Worsening**	
	***n***	**%**	***n***	**%**	***n***	**%**	***n***	**%**
Headache	41	37.3	31	28.2	71	64.5	8	7.3
Vomiting	13	11.8	12	10.9	92	83.6	6	5.5
Visual impairment	34	30.9	14	12.7	91	82.7	5	4.5
Seizures	19	17.3	16	14.5	92	83.6	2	1.8
Motor deficits	76	69.1	35	31.8	64	58.2	11	10.0
Sensory deficits	60	54.5	31	28.2	71	64.5	8	7.3
NFS 0	4	3.6	0	0.0	3	2.7	1	0.9
NFS 1–2	83	75.5	32	29.1	31	28.2	20	18.2
NFS 3–4	23	20.9	7	6.4	10	9.1	6	5.5

*NFS 0: asymptomatic, NFS 1–2: mild or moderate neurologic symptoms, NFS 3–4: severe neurologic symptoms, requiring hospitalization*.

## Discussion

We have conducted a retrospective analysis of 110 patients with LC, treated with palliative radiotherapy at our institution over a 10-year period. Treatment was well-tolerated and achieved a palliative effect regarding symptom stabilization or even improvement in the majority (75.5%) of patients. Overall survival at 13.9 weeks was comparable to figures published in recent literature for similar patient cohorts, as will be discussed. We were able to identify initial clinical performance and symptom response to treatment as favorable and elevated LDH levels as unfavorable independent prognostic factors for OS.

High-level evidence on the treatment of LC is scarce, and most current clinical guidelines are largely based on small retrospective series or expert consensus (1, 32). One reason is the relative rarity of LC diagnosis and until recently, the exclusion of patients with LC from most prospective clinical trials (33). The current EANO-ESMO as well as the NCCN guidelines recommend a multidisciplinary approach with regard to diagnostics and treatment of patients with LC (1, 32). This approach is based on exact MRI imaging, complemented by pathological confirmation in CSF cytology (34–36). Radiotherapy is recommended in the form of WBRT to treat additional brain metastases and symptomatic intracranial lesions. Furthermore, focal spinal irradiation is performed to alleviate pain and neurologic symptoms and in the case of nodular disease to restore CSF flow (37). Regarding medicamentous therapy, systemic and intrathecal chemotherapy application have previously shown efficacy (38–40). Recently, molecularly targeted substances such as tyrosine kinase inhibitors (TKI) have produced encouraging results with proven activity in the CNS (41). In this context, primary histology and molecular analyses play an important role in recent data, since they decisively influence the range of available substances for systemic and LC treatment (33, 42, 43).

For patients with CNS metastases from breast cancer, commonly employed systemic cytotoxic agents include methotrexate (MTX) and 5-fluorouracil (5-FU) (1, 44). Trastuzumab has yielded promising results in patients with HER2-positive tumors, although evidence on the efficacy of trastuzumab-emtansine (T-DM1) is limited (45, 46). Regarding the use of TKI, lapatinib might be a promising novel agent when combined with capecitabine (47, 48). Several recent analyses have retrospectively evaluated the outcome of reasonably sized cohorts of between 60 and 100 patients (11, 21, 33). Those patients most commonly received a combination of radiotherapy and ITC with methotrexate, thiotepa or liposomal cytarabine. Estimated OS ranged from 10 to 17 weeks, and the application of ITC, as well as the combination with systemic chemo- or antihormonal therapy, was prognostic for a favorable outcome. On the other hand, high-grade histology, poor clinical performance and triple-negative tumors had a significant negative impact on survival (11, 43, 49).

Several retrospective analyses of cohorts sized between 100 and 150 patients evaluated survival in patients with LC from NSCLC. OS estimates in those series ranged between 13 and 15 weeks and were thus similar to those previously discussed for breast cancer (8, 12, 50). Favorable prognostic impact was detected for the use of ITC, WBRT, and concurrent chemotherapy, while poor clinical performance and uncontrolled intracranial pressure yielded inferior OS (8, 12, 50). Notably, patients who received EGFR-TKI therapy showed a median OS of up to 14 months in several cohorts (12, 51). Molecularly targeted substances have played a role of rising importance in the treatment of CNS metastases in patients with correspondingly mutated adeno-NSCLC. Recent developments on this horizon have been comprehensively reviewed by Cheng and colleagues (42). Although most of the reviewed data featured only small retrospective cohorts of up to 35 patients, results were conclusive in demonstrating superior OS for EGFR- or anaplastic lymphoma kinase (ALK)-mutated patients receiving corresponding TKI therapy when compared to non-mutated patients. Median OS in those cohorts ranged from 3.5 to 12 months. Prospective evidence regarding CNS response is currently emerging for newer TKIs such as osimertinib: In the AURA extension trial, median duration of CNS response was 15.2 months and progression-free survival (PFS) was 12.3 months for patients with brain metastases receiving osimertinib. A recent subgroup analysis of the FLAURA trial reported a CNS PFS rate of 77% after 12 months for patients with brain metastases and an objective response rate of 60% for a smaller subgroup of 5 patients with radiological LC, treated with osimertinib (52). Several smaller case series have reported similarly promising efficacy in patients with LC, achieving a median OS of up to 18 months (53, 54).

The primary role of radiotherapy for LC patients is symptom control and the treatment of bulky or nodular disease (1, 55). The more extensive approach of craniospinal irradiation aims at tumor cell eradication in all CNS compartments and is frequently employed for the treatment of pediatric and primary CNS tumors such as medulloblastomas or ependymomas (30, 56). Its role in the treatment of LC is marginal due to the substantial toxicity, particularly hematologic. Modern irradiation techniques, such as intensity-modulated radiotherapy with helical dose delivery or proton therapy have allowed for a significant reduction of the toxicity caused by CSI (30, 57, 58). Nevertheless, the scarce evidence available on this approach suggests, only few and highly selected patients with good clinical performance might benefit from CSI for the treatment of LC (20, 59). Our findings concerning the 25 patients, who received CSI in the current collective, confirm this rationale, although OS in this subgroup did not significantly differ from the rest of the collective and the occurrence and intensity of treatment-associated toxicity was not elevated in comparison. Regarding only the CSI-subgroup, younger age at LC diagnosis, good clinical performance and symptom response to treatment were prognostic of superior OS. Detailed clinical and technical aspects of this subgroup and an in-depth analysis of factors possibly relevant for patient selection have been discussed separately (60).

Regarding the efficacy of WBRT, several reports have found it to be associated with prolonged survival (12, 13), while other reports, as well as our current data could not show a statistically significant effect on OS (8). However, in our collective, WBRT was primarily performed in patients with additional symptomatic brain metastases and poor clinical performance, with those strong clinical prognosticators largely defining oncologic outcome, irrespective of WBRT. On the other hand, we could achieve a stabilization of intracranial pressure-associated symptoms such as headaches and vomiting in up to 83.6% of the patients and an improvement in up to 28.2%, emphasizing the palliative value of WBRT in LC patients. Neurologic function, quantified in the form of the NFS score, has proven a useful tool in the current analysis, providing better comparability and facilitating the assessment of treatment-induced effects. Two past RTOG trials, as well as several recent publications have confirmed the role and applicability of the NFS for the clinical assessment of patients with CNS metastases (22–24). Like any form of purely clinical assessment, the NFS is to a certain extent prone to subjectivity and inter-observer variability. While this may limit its applicability as an objective outcome measure, it is a tradeoff in return for simplicity and ease of clinical use. In general, however, structured analyses on the functional outcome and palliative effect of RT in LC patients are scarce, and no comparable works providing an assessment of individual symptoms as well as general neurologic function are available.

Patient selection is evidently crucial in the context of LC to avoid the burden of over-treatment in a highly palliative situation with severely limited prognosis: The body of literature discussed above widely agrees on the prognostic value of initial clinical performance, represented by the KPI (12–14, 49, 61, 62). We could confirm the impact of this prognosticator in our analysis, while additionally providing a quantitative functional assessment in the form of the NFS. Statistical significance in multivariate analysis was not reached for the initial NFS score, possibly due to correlation with the KPI. However, NFS outcome in response to treatment was prognostic for overall survival, potentially aiding in the decision about the pursuit or omission of further oncologic therapy after palliative RT. A high tumor burden, represented by strongly elevated serum LDH levels, was prognostic of inferior OS. Consequently and in light of the prospective evidence available on brain metastases, LC patients with low performance status and high tumor burden might be candidates for treatment limitation to corticosteroids and best supportive care (63).

Histology had no significant impact on survival in our current collective, for which several reasons are conceivable. As discussed above, with the availability of new histology-dependent and molecularly targeted therapies, oncologic prognosis is critically influenced by medicamentous treatment, while the focus of radiotherapy lies on symptom control (41). Accordingly, prolonged survival may be observed in histologies for which efficacious substances are available, such as breast cancer and adeno-NSCLC (42, 43). As the patients in our current collective have been treated over a period of 10 past years, only a minority received treatment with newer substances for which a substantial impact on survival can be expected. Additionally, only a small fraction of the analyzed patients received any systemic therapy in combination with or after the completion of RT. For this subgroup, a significant survival benefit could be shown, although it comprised tumors of different histologies.

In the context of LC, the safety of combining novel targeted substances with palliative radiotherapy is a question of rising importance. A recently published meta-analysis examined seven studies including patients with brain metastases, who received sequential or simultaneous TKI treatment and radiotherapy to the CNS (WBRT or stereotactic irradiation). No significant increase in neurotoxicity was observed for erlotinib and gefitinib (64, 65). Data on newer substances such as afatinib or osimertinib is scarce at best, consisting of small series or case reports (66–68). Reliable data regarding the safety and efficacy of combined treatment might in recent future prove of great importance in the treatment of LC, where fast and effective symptom palliation is just as crucial as an uninterrupted administration of effective systemic therapy (41, 68).

Limitations of the current study include its retrospective nature and relatively small number of patients. Furthermore, the heterogeneity of the patient collective, regarding histology and systemic treatment, makes it difficult to reliably identify treatment-related factors with a significant impact on survival. The fraction of patients receiving LC treatment with novel, molecularly targeted agents at 12.7% was relatively small, limiting the extent to which those treatments' effects on survival can be assessed. However, the palliative value of radiotherapy, providing symptom control or improvement in the majority of cases was evident by the current data and should be considered independently of overall prognosis.

## Conclusion

To the best of our knowledge, this is the largest cohort in current literature to focus on the palliative efficacy of radiotherapy in LC patients of different histologies, while providing detailed analysis of symptom control and neurologic function. We could identify initial clinical performance, symptom response to treatment and serum LDH levels as independent prognostic factors for survival. Although general prognosis nowadays is decisively influenced by the availability of efficacious systemic treatment, radiotherapy is invaluable in providing effective palliation of neurological symptoms. Due to the limited life expectancy associated with LC, patient selection is mandatory to avoid over-treatment.

## Data Availability

The datasets used and/or analyzed during the current study are available from the corresponding author on reasonable request.

## Author Contributions

RE, DB, JD, and SR planned and supervised this analysis as part of the neuro-radiooncological research group. KB performed data collection and review. DW performed all statistical analysis. RE reviewed data analysis and drafted the manuscript. KL, FS, SA, AP, MH, SK, JH-R, and PH contributed patient data and participated in reviewing and improving analysis and manuscript. SL provided neurologic review and guidance on respective aspects. All authors read and approved the final manuscript.

### Conflict of Interest Statement

The authors declare that the research was conducted in the absence of any commercial or financial relationships that could be construed as a potential conflict of interest.
